# A Novel Bayesian Approach for EEG Source Localization

**DOI:** 10.1155/2020/8837954

**Published:** 2020-10-30

**Authors:** Vangelis P. Oikonomou, Ioannis Kompatsiaris

**Affiliations:** Information Technologies Institute, CERTH, Thessaloniki, Greece

## Abstract

We propose a new method for EEG source localization. An efficient solution to this problem requires choosing an appropriate regularization term in order to constraint the original problem. In our work, we adopt the Bayesian framework to place constraints; hence, the regularization term is closely connected to the prior distribution. More specifically, we propose a new sparse prior for the localization of EEG sources. The proposed prior distribution has sparse properties favoring focal EEG sources. In order to obtain an efficient algorithm, we use the variational Bayesian (VB) framework which provides us with a tractable iterative algorithm of closed-form equations. Additionally, we provide extensions of our method in cases where we observe group structures and spatially extended EEG sources. We have performed experiments using synthetic EEG data and real EEG data from three publicly available datasets. The real EEG data are produced due to the presentation of auditory and visual stimulus. We compare the proposed method with well-known approaches of EEG source localization and the results have shown that our method presents state-of-the-art performance, especially in cases where we expect few activated brain regions. The proposed method can effectively detect EEG sources in various circumstances. Overall, the proposed sparse prior for EEG source localization results in more accurate localization of EEG sources than state-of-the-art approaches.

## 1. Introduction

Brain imaging techniques are important tools since they give us the ability to understand the neural mechanisms of complex human behavior in cognitive neuroscience. Also, they have clinical applications in patients with brain tumors and epilepsy where functional brain imaging is useful for neurosurgical planning and navigation [1–4]. Among various brain imaging techniques, electroencephalography (EEG) is preferable due to the low cost of EEG devices, the high temporal resolution of EEG signal, and the portability of EEG devices. The EEG is a noninvasive brain imaging technique that measures the scalp electric potentials produced by the firing of a very large number of neurons functioning inside the brain. The identification of firing neurons is very crucial since it gives us the ability to study brain dynamics in time scales of milliseconds. The identification of the electric current sources responsible for the electrical activity inside the brain based on the EEG activity recorded at the scalp (through electrodes) is one of the major problems in EEG processing. This problem is referred to as the EEG source localization [3, 4] or EEG inverse problem [3, 5].

The EEG inverse problem involves the calculation of locations and amplitudes of EEG sources given the EEG activity and the geometry and conductivity properties of the head. During the last two decades, a wide range of methods have been developed for the identification of EEG sources. These can be classified into two large groups: (a) dipole-fitting models and (b) distributed-source models. Dipole-fitting models represent the brain activity using a small number of dipoles and try to estimate the amplitudes, the orientations, and the position of a few dipoles that explain the data [4, 5]. However, these methods are sensitive to the initial guess of the number of dipoles and their initial locations. On the other hand, distributed-source methods use a large number of dipoles with fixed positions and try to estimate their amplitudes by solving a linear inverse problem [4, 5]. The EEG linear inverse problem is ill-posed since the number of EEG sources is much larger than the number of EEG sensors. Also, the problem is becoming more difficult due to the presence of noise.

The distributed-source methods can be divided into two large families, reflecting how they deal with the dimension of time. From one side, we have methods that estimate the spatial source distribution instant by instant [3], while on the other side, we have the spatiotemporal modelling approaches [3, 4]. Both families have their advantages and disadvantages. For example, instant-by-instant (or instantaneous) methods are suitable for continuous brain scanning [3], while spatiotemporal methods are suitable for EEG sources with oscillatory activity [3]. Among the first reported instantaneous methods is that of the Minimum Norm Estimation (MNE) [6]. However, this method tends to prefer low-activity EEG sources close to the surface over strong-activity EEG sources in depth. To correct this problem, various methods have been proposed including weighted minimum norm, Loretta [7] and sLoretta [8]. The above methods need to adjust the regularization parameter through a cross-validation procedure or the L-curve method [5]. To account for the time evolution of an EEG source, authors have used spatiotemporal models [4, 9, 10]. Representative algorithms of this family are the Multiple Sparse Priors algorithm [11], the Champagne algorithm [12], and algorithms based on the Kalman Filtering [9]. Assuming that we have much larger time points than sensors, these algorithms provide us with accurate estimates on how a source evolves across time.

EEG sources could possess various properties related to the induced brain activity. For an EEG source, it is critical to know if it is focal or not [13, 14], its spatial pattern (how its neighborhood is affected) [11, 15, 16], and if the oscillatory activity is present or not across time [3, 9–11, 15]. Furthermore, a combination of EEG sources produces complex brain activity that spans across multiple spatial (and/or time) scales [15]. All these properties could be observed either in conjunction or in disjunction depending on the underlying EEG study. Furthermore, these properties are included in the overall analysis through the assumed EEG sources' model and various assumptions about the model. Clearly, the linear observation model [17, 18], the linear dynamical model (or Kalman Filters) [17, 18], and the multiple measurement vector (MMV) model [19] make different generative modelling assumptions about the underlying mechanisms that produce the EEG data.

The spatial properties of EEG sources are encoded into the linear observation model through the use of prior distributions or regularization terms. In cases where we expect localized activity (i.e., in certain types of epilepsy), a suitable assumption is to assume that EEG sources are sparse, meaning that a few of them are activated at a specific time instant. In that case, sparse prior distributions could be used [13] or regularization terms in the form of L1-norm [14, 20]. However, EEG sources can also be both sparse and spatially distributed. Based on that, many authors develop various sparsity-promoting methods by including in their method the spatially diffused property by segmenting the brain into different predefined regions [11], by using regularization terms that take into account the spatial extension of EEG sources [21], by extending the lead field matrix to multiple spatial scales [15, 16]. However, the spatial scale over which sparsity might apply remains an area of investigation.

In the present work, we propose a new framework to deal with localized (focal) activity, which can be extended in multiple spatial scales. Our contributions, with respect to the EEG source localization, are (a) a new sparse prior for the localization of EEG sources [22] and its extension to include group-sparse structures, (b) an extended (or modified) lead field matrix for the case of spatially extended EEG sources, and (c) extensive experiments using three real EEG datasets with various properties and differences between them. A preliminary version of this work has been reported in [22]. The remainder of this paper is organized as follows. In Section 2, we describe the proposed algorithmic approach for the solution of the inverse EEG problem. Then in Section 3, we present the experiments of our approach on synthetic and real EEG data. Also, a comparison of our algorithms with baseline and state-of-the-art algorithms is provided. Finally, in Section 4, we discuss our conclusions and future directions of our work.

## 2. Materials and Methods

### 2.1. Linear Observation Model

In EEG inverse problem, we desire to find the brain activity given the EEG measurements and the geometry and conductivity properties of the head. In our work, we use the distributed-source model. This means that we use a finite number of dipoles in the cortex at given locations. Hence, the potential at the scalp is a linear combination of dipoles amplitudes, represented by the following equation:

(1) $$\begin{array}{c} y=Hx+e, \end{array}$$

where **y** ∈ *ℜ*^*N*^ is the EEG measurement vector acquired by the *N* electrodes, **x** ∈ *ℜ*^3*M*^ contains the amplitudes of *M* dipoles along the three spatial dimensions, and **H** ∈ *ℜ*^*N*×3*M*^ is the lead field matrix that describes the propagation of electromagnetic field from the sources to the sensors and it contains information related to the geometry and conductivity properties of the head. The vector **e** is an additive white Gaussian noise. The EEG inverse problem of the observation model of equation (1) consists of estimating the vector **x** given the data **y** and the lead field matrix **H**. In the next subsection, we describe an approach for this process by using the variational Bayesian (VB) framework. More specifically, we define the hierarchical sparse prior over the amplitudes of EEG sources, the likelihood of the model, and its hyperparameters. Also, we can observe here that our instantaneous linear observation model is suitable for cases where we do not have a correlation between time samples, the noise, and sources which are nonstationary quantities, and the number of time samples is smaller than the number of sensors.

Distributed EEG source localization represents a highly ill-posed problem since the measurements are in order of 10^2^ while unknowns are in order larger than 10^4^. One approach to reducing the complexity of the problem is to restrict the solutions space by reducing the number of unknowns. In this direction, two approaches are used considerably: the restriction of solutions (or EEG sources) to the cortical surface of the brain and the placement of constraints in dipole orientation [23, 24]. The above restrictions are reflected in the construction of the lead field matrix **H**. In our work, we examine both the aforementioned cases.

### 2.2. Sparse Bayesian Learning

From a machine learning perspective, sparsity is a very helpful property since the processing is faster in a sparse representation where few coefficients reveal the information we are looking for. Hence, sparse priors help us to determine the model order in an automatic way and reduce its complexity. In addition to the above, from a brain imaging perspective, the motivation of using sparse priors is based on the localized (or focal) activity that can be observed in certain types of epilepsy and on observed sparse activations in the brain during high cognitive processing as revealed by various brain imaging techniques. In [13], sparse priors, based on a Bernoulli Laplacian prior, are used resulting in a posterior distribution where the estimators cannot be computed with close-form expressions. For this reason, the authors in [13] use the Markov Chain Monte Carlo framework.

In this work, the EEG sources **x** are treated as a random variable following a Gaussian distribution of zero mean and variance *a*_*i*_^−1^*λ*_*i*_^−1^:

(2) $$\begin{array}{c} p\left(x|a;\lambda \right)=N\left(x|0,\Lambda \right)=\prod_{i=1}^{3M}N\left(x_{i}|0,a_{i}^{-1}\lambda_{i}^{-1}\right), \end{array}$$

where *𝒩* is the symbol for Gaussian distribution. In Sparse Bayesian Learning literature [18, 25, 26], a common approach is to assume that the covariance matrix Λ is a diagonal matrix with elements *a*_*i*_^−1^, *i*=1,…, 3*M*. Each parameter *a*_*i*_, which controls the prior distribution of the EEG sources **x**, follows a Gamma distribution, so the overall prior over all *a*_*i*_ is a product of Gamma distributions given by *p*(**a**)=∏_*i*=1_^3*M*^Gamma(*a*_*i*_; *b*_*a*_, *c*_*a*_). However, in our study, we introduce one more parameter into the distribution. More specifically, we assume that the covariance matrix Λ is a diagonal matrix with elements *a*_*i*_^−1^*λ*_*i*_^−1^, *i*=1,…, 3*M*. In our analysis, parameters *λ*_*i*_ are assumed to be known and deterministic quantities.

At this point, it is worth examining the marginal prior distribution of EEG source *x*_*i*_ by eliminating the hyperparameters *a*_*i*_:

(3) $$\begin{array}{c} p\left(x_{i};\lambda_{i}\right)=\int p\left(x_{i}|a_{i};\lambda_{i}\right)p\left(a_{i}\right)\text{d}a_{i}=\int N\left(x_{i}|0,a_{i}^{-1}\lambda_{i}^{-1}\right)\text{Gamma}\left(a_{i};b_{a},c_{a}\right)\text{d}a_{i}\\ \propto {\left(\frac{\lambda_{i}}{b_{a}}\right)}^{1/2}{\left[1+\frac{\lambda_{i}x_{i}^{2}}{b_{a}}\right]}^{-\left(c_{a}+1/2\right)}. \end{array}$$

Equation (3) can be recognized as a Student-*t* distribution with zero mean, shape parameter *c*_*a*_, and scale parameter *b*_*a*_/*λ*_*i*_. We can see that parameter *λ*_*i*_ controls the scale of the Student-*t* distribution. In addition, by adopting a procedure similar to [25], we can show that the EEG sources have the improper prior *p*(*x*_*i*_) ∝ 1/(*λ*_*i*_^1/2^ · |*x*_*i*_|). Now, by setting *λ*_*i*_⟶1/|*x*_*i*_|, we obtain *p*(*x*_*i*_) ∝ 1/·|*x*_*i*_|^1/2^ which can be recognized as an extremely “sparse” prior.

The overall precision (inverse variance) *β* of the noise follows a Gamma distribution: *p*(*β*)=Gamma(*β*; *b*, *c*)=(1/(Γ(*c*)))((*β*^(*c* − 1)^)/*b*^*c*^)exp{−*β*/*b*}, where *b* and *c* are the scale and the shape of the Gamma distribution, respectively. We use the Gamma distribution for the noise components for two reasons: first, this distribution is conjugate to the Gaussian distribution, which helps us in the derivation of closed-form solutions, and second, it places the positivity restriction on the overall variance and the scaling parameters.

So, the overall prior over model parameters {**x**, **a**, *β*} is given by *p*(**x**, **a**, *β*; *λ*)=*p*(**x***| ***a**; *λ*)∏_*i*=1_^3*M*^*p*(*a*_*i*_)*p*(*β*). The likelihood of the data is given by

(4) $$\begin{array}{c} p\left(y|x,\beta ;\lambda \right)=\frac{\beta^{N/2}}{{\left(2\pi \right)}^{N/2}}\cdot \text{exp}\left\{-\frac{\beta }{2}{\left(y-Hx\right)}^{T}\left(y-Hx\right)\right\}. \end{array}$$

To apply the VB methodology [17], we need to define an approximate posterior based on one factorization over the parameters {**x**, **a**, *β*}. In our study, we choose the following factorization: *q*(**x**, **a**, *β*; *λ*)=*q*(**x***| ***a**; *λ*)∏_*i*=1_^3*M*^*q*(*a*_*i*_)*q*(*β*).

Applying the VB methodology and taking into account the above factorization, the following posteriors are obtained:

(5) $$\begin{array}{c} q\left(x\right)=N\left(\hat{x},C_{x}\right),\\ q\left(\beta \right)=\text{Gamma}\left(\beta ;b^{\prime },c^{\prime }\right),\\ q\left(a\right)=\prod_{i=1}^{D}\text{Gamma}\left(a_{i};b_{a_{i}}^{\prime },c_{a_{i}}^{\prime }\right). \end{array}$$

The moments of each distribution are calculated by applying iteratively the following equations until convergence:

(6) $$\begin{array}{c} C_{x}^{\left(k+1\right)}={\left({\hat{\beta }}^{\left(k\right)}H^{T}H+{\hat{\Lambda }}^{\left(k+1\right)}\right)}^{-1}, \end{array}$$

(7) $$\begin{array}{c} {\hat{x}}^{\left(k+1\right)}={\left({\hat{\beta }}^{\left(k\right)}H^{T}H+{\hat{\Lambda }}^{\left(k+1\right)}\right)}^{-1}\hat{\beta }H^{T}y, \end{array}$$

(8) $$\begin{array}{c} \frac{1}{b_{a_{i}}^{{\left(k+1\right)}^{\prime }}}=\frac{\lambda_{i}^{\left(k+1\right)}}{2}\left({\left({\hat{x}}_{i}^{\left(k+1\right)}\right)}^{2}+C_{x}^{\left(k+1\right)}\left(i,i\right)\right)+\frac{1}{b_{a}}, \end{array}$$

(9) $$\begin{array}{c} c_{a_{i}}^{{\left(k+1\right)}^{\prime }}=\frac{1}{2}+c_{a}, \end{array}$$

In the above equations, the matrix ${\hat{\Lambda }}^{\left(k+1\right)}$ is a diagonal matrix with ${\hat{a}}_{i}^{\left(k\right)}\cdot \lambda_{i}^{\left(k+1\right)}$ in its main diagonal. For *λ*_*i*_^(*k*+1)^, we follow the considerations of [27] and we set them to $1/\left|{\hat{x}}_{i}^{\left(k\right)}\right|$. With respect to other similar approaches [25, 28], we can observe the difference in equations (7) and (8). More specifically, in our approach, the parameter *b*_*a*_*i*__′ is weighted by the corresponding parameter *λ*_*i*_. Observe here that this parameter is affecting the scale of marginal Student-*t* distribution (see equation (3)).

### 2.3. Group-Sparse Priors

In the subsequent analysis, we assume that the EEG sources **x** have a group structure. More specifically, we define *G* groups of EEG sources such that the vector **x**_*g*_ contains *d*_*g*_ coefficients assigned to group *g*. Sparsity between groups can be achieved by selecting carefully the prior distribution over them. Assuming a priori independence between groups and that each group follows a Gaussian distribution with zero mean and covariance matrix *a*_*g*_^−1^**I**_*d*_*g*__, the prior over coefficients is given by

(10) $$\begin{array}{c} p\left(x|a\right)=\prod_{g=1}^{G}N\left(x_{g}|0_{d_{g}},a_{g}^{-1}I_{d_{g}}\right), \end{array}$$

where *𝒩* is the symbol for Gaussian distribution. Furthermore, we assume that each parameter *a*_*g*_, which controls the group sparsity of the EEG sources **x**, follows a Gamma distribution, so the overall prior over all *a*_*g*_ is a product of Gamma distributions given by *p*(**a**)=∏_*g*=1_^*G*^Gamma(*a*_*g*_; *b*_*a*_, *c*_*a*_). The above hierarchical prior belongs to the family of conjugate distributions and it is well known for its sparse properties [25, 26] with respect to the groups. As before (see Section 2.2), we change the above prior by introducing one more parameter. More specifically, we assume that the prior covariance matrix is a diagonal matrix with elements *a*_*g*_^−1^*λ*_*g*_^−1^. In our analysis, parameters *λ*_*g*_ are assumed to be known and deterministic quantities. Now, the prior distribution of coefficients is given by

(11) $$\begin{array}{c} p\left(x|a;\lambda \right)=\prod_{g=1}^{G}N\left(x_{g}|0,a_{g}^{-1}\lambda_{g}^{-1}I_{d_{g}}\right). \end{array}$$

Using the above group-sparse prior and following a similar VB procedure as that in the previous section, we can derive an iterative algorithm. More information about the derivation of the group-based algorithm can be found in [29]. Also, with respect to the above algorithm, a coefficient could potentially belong to several groups. Overlapping between groups is permitted; however, special care must be taken in order to reflect the anatomical and functional properties of the brain.

It interesting at this point to examine possible group strategies with respect to the inverse EEG problem. We can observe here that in equation (1) each dipole is represented by three components in the lead field matrix, one for each of the three spatial dimensions. So, an obvious choice of grouping is to define one group for each dipole. In that case, we have *G*=*M* and *d*_*g*_=3 for the group-sparse prior. Another choice of grouping is to use an anatomical (or functional) template (or brain maps) to define the groups. Finally, a third option is to define the groups by using a criterion based on distances between the dipoles (i.e., dipoles in close distance are expected to behave in a similar fashion). Observe here that the first two group creation strategies are based on information related to the brain's structure, organization, and function. Also, in these cases, one dipole belongs only to one group (the groups are disjointed sets and there is no overlap between them), while in the distance-based grouping, one dipole could belong to various groups (overlapping between groups exists). In all the above cases, the structure of groups is considered known before the application of the algorithm.

### 2.4. Spatially Extended EEG Sources

In the above sections, we have assumed that the EEG sources are focal in nature and we examined their sparseness in the original EEG source domain. However, EEG sources could be spatially extended in cases such as in cognitive tasks or spontaneous states [3]. In this subsection, we borrow one of the general ideas from the Compress Sensing framework [30]. More specifically, we assume that EEG sources, **x**, are sparse in another domain which we call it *ψ*-domain. In our approach, the *ψ*-domain could be the wavelet domain, Fourier domain, discrete cosine domain, or any other linear transformation and it is represented by the matrix Ψ. The EEG sources, **x**, can be written as

(12) $$\begin{array}{c} x=\Psi z, \end{array}$$

where **z** is a vector that contains the coefficients of EEG sources in the *ψ*-domain and also this vector has sparse nature due to the assumption of sources' sparseness in the *ψ*-domain. Now, the basic equation of our work (equation (1)) can be written in the *ψ*-domain as

(13) $$\begin{array}{c} y=Hx+e=H\Psi z+e=H_{\psi }z+e. \end{array}$$

We can observe here that the original lead field matrix has been modified by the transformation matrix Ψ, **H**_*ψ*_=**H**Ψ. Using one of the previous algorithms (or any other sparsity induced algorithm), we can find the coefficients **z**, and finally, the EEG sources can be obtained by using equation (12).

The choice of *ψ*-domain (which is reflected in the structure of matrix Ψ) is crucial for the properties of original EEG sources, **x**. Also, this choice must incorporate some prior knowledge about the original EEG sources. Observe here that the EEG sources are positioned on a grid in 3D space; hence, direct use of wavelet transform or Fourier transform is not an easy task. Furthermore, interpretation of the results from a neurophysiological viewpoint is more difficult. Since our goal is to find spatially extended EEG sources, we adopt a local spatial smoothing kernel [16]. More specifically, for *i*-th EEG source, we define

(14) $$\begin{array}{c} \psi_{ij}=\left\{\begin{array}{cc} 1, & \text{if }i=j,\\ \text{exp}\left(-r\cdot d_{ij}^{2}\right), & \text{if }i\neq j, \end{array}\right. \end{array}$$

where *d*_*ij*_, *i*=1,…, *N*, *j*=1,…, *N* is the spatial distance between the *i*-th and *j*-th EEG sources, while *r* is a parameter that controls the extension of spatial smoothness between individual EEG sources. In our work, the parameter *r* is assumed to be known; however, we can estimate it by using a cross-validation approach or methods based on multiple kernel learning [31, 32]. Looking at equation (12), we can verify that the original EEG sources are spatially extended due to Ψ**z** and the properties of the vector **z** (sparsity) and the matrix Ψ (spatially extended).

Concluding this section, we want to mention that three approaches, using the Bayesian framework, are provided. The first approach (we call it *Fan*) is described in Section 2.1 and it presents the backbone of our overall methodology. This method is suitable for finding focal EEG sources due to its sparse properties. The second approach (we call it *FanGr*) is an extension of *Fan* approach. The main characteristic of this method is that now we can define groups over EEG sources. Finally, the third approach (we call it *FanSmooth*) is similar to the first approach but with one critical difference in the lead field matrix. In this last approach, we use a modified lead field matrix using ideas from CS framework.

## 3. Experiments and Results

In this section, we present our experiments with the corresponding results using synthetic EEG data and real EEG data from three EEG experiments. The real EEG data are produced due to the presentation of auditory and visual stimulus on the participants. In all our experiments, we have used the FieldTrip toolbox [33] to preprocess the EEG data and to construct the lead field matrices. In our study, we adopted two approaches for the construction of lead field matrices, the cortical-based approach, and the volumetric-based approach.

### 3.1. Experiments Using Synthetic EEG Data

Synthetic data with few pointwise source activations (see equation (1)) were generated using realistic head models with electrodes placed according to the 10–10 international system of electrode placement. In our study, we investigate two cases of, with respect to the number of channels, 128 channels and 256 channels.

#### 3.1.1. Activations

In our work, we investigated two different kinds of activations: (1) single dipole activations, and (2) multiple dipole activations. The first case represents a situation where one dipole is activated among many, and the second case represents a situation where many dipoles (possibly distant) are activated. The amplitudes of active EEG sources were samples from a Gaussian distribution with zero mean and variance one. Finally, with respect to EEG measurements, we examine two cases: noise-free measurements and noisy measurements. In noisy measurements, we added white Gaussian noise and the signal-to-noise ratio (SNR) was defined to 60 dB.

#### 3.1.2. Lead Field Matrix

With respect to the lead field matrix, we examined two cases for its construction: the cortical-based case and the volumetric-based case. *Cortical based*: in this case, the dipoles are placed on a spatial grid covering the cortical surface. The positions and orientations of dipoles are fixed. In addition, orientations are normals to the cortical surface [13, 24]. Finally, from the perspective of neurophysiology, the source space is the cortex (i.e., we assume that the observed electrical activity is produced by a specific brain structure). The number of dipoles was 5124; hence, the resulting lead field matrix is **H** ∈ *ℜ*^128×5124^ or **H** ∈ *ℜ*^256×5124^. *Volumetric (or grid) based*: in this case, the dipoles are placed on a spatial grid covering the entire brain. Also, the positions of dipoles are fixed but the orientations are free. In addition, the source space includes the cortex, subcortical structures, and the cerebellum. The grid resolution was set to 1 cm resulting in 2020 dipoles; hence, the resulting lead field matrix is **H** ∈ *ℜ*^128×6060^ or **H** ∈ *ℜ*^256×6060^. Overall, in this set of experiments, we examine configurations of inverse EEG problems with respect to the number of channels, the type of lead field matrix, the presence (or not) of noise, and the type of activations. Each configuration is repeated 50 times in order to obtain averaged results with respect to the performance of each method.

#### 3.1.3. Performance Measures

In order to evaluate the performance of an algorithm, we adopt the following measures. *Reconstruction error*: we use the reconstruction error between the true EEG sources, **x**_true_, and the estimated EEG sources, **x**_est_, given by ‖**x**_est_ − **x**_true_‖_2_^2^/‖**x**_true_‖_2_^2^. This measure will determine whether the algorithm recovers the source energy. *Localization error* [20]: we use the Euclidean distance between the simulated source and the maximum of the estimated activity within the sphere neighboring the simulated source. This measure will determine whether the algorithm is able to find the point of the simulated source. In our study, the neighbor was set to 25 mm [20]. *A*′*metric* [16]: this metric is computed as *A*′=((*H*_*R*_ − *F*_*R*_)/2)+(1/2), where *H*_*R*_ is the hit rate and *F*_*R*_ is the false positive rate. This measure estimates the area under the Receiver Operator Characteristic (ROC) curve and it is related to the detection accuracy of the algorithm (if the area under the ROC is large, then the hit rate is high compared to the false positive rate). In order to define the hit rates, we follow a similar procedure to that of [16], where we included in the calculation of hit rates voxels that are at least 0.1% of the maximum activation of the localization result. Finally, we compared our methods with the following approaches: (a) the Minimum Norm Estimator (*MNE*) [4, 6], a classical approach for the EEG inverse problem, (b) the Relevance Vector Machines using the VB approach (*RVM-VB*) [28], and (c) the plain Champagne (*Champ*) [4, 12] using the available code from the NUTMEG toolbox [34].

#### 3.1.4. Results on Synthetic EEG Data

**Figure 1:**
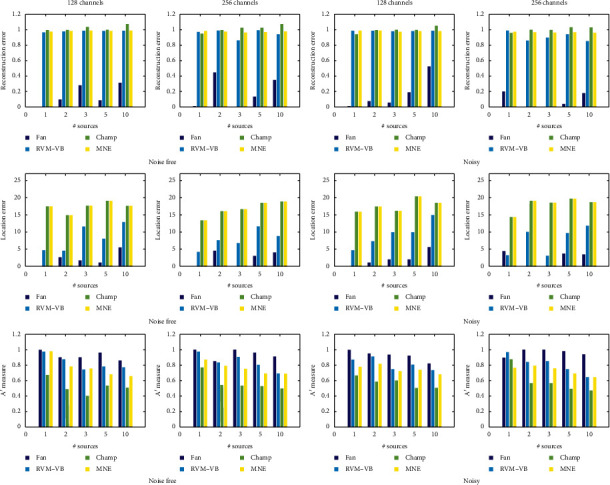
Obtained performance measures in the case of cortical-based lead field matrix.

**Figure 2:**
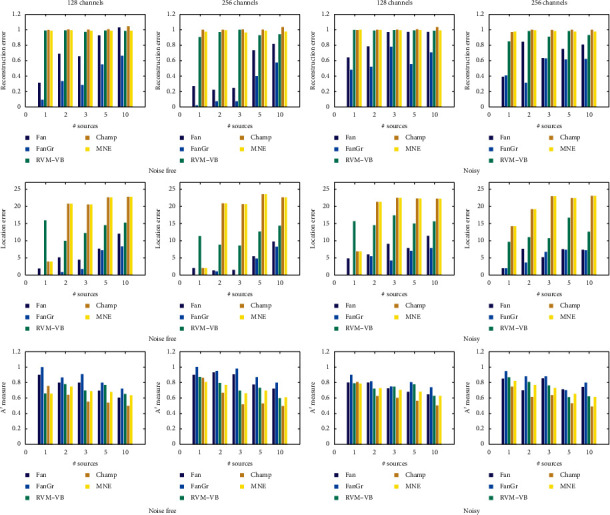
Obtained performance measures in the case of volumetric-based lead field matrix.

In Figure 1, we provide the obtained results when a cortical-based lead field matrix is used with respect to all performance measures. The results are shown with respect to the measures, the number of active EEG sources, the number of channels, and the presence (or not) of noise. We can see that the proposed approach presents the best performance compared to other methods. More specifically, the proposed method presents the smallest reconstruction and location error and the highest value for *A*′ metric. This is observed in all cases irrespective of the number of active EEG sources or the number of channels or to the presence of noise. Additionally, in Figure 2, we present the obtained results when the volumetric-based lead field matrix is used. In this set of experiments, we use, also, the group version of our method since one dipole can be considered as a group of three elementary dipoles (one for each of three spatial dimensions). We observe that both versions of our approach present better performance (in terms of reconstruction error, location error, and *A*′ metric) than the other methods. Also, we can see that, for the majority of activation profiles, the adoption of grouping structures increases the performance of our analysis, especially when we have multiple activations. Clearly, the proposed approach is able to reconstruct more accurately the spatial pattern of EEG sources without introducing error in the location of EEG source(s) resulting in high detection accuracy.

### 3.2. Experiments Using Real EEG Data

In this section, we provide our results from experiments using real EEG data from three EEG datasets. The EEG experiments were designed to study brain responses with respect to auditory and visual stimuli. Furthermore, in this section, we include in our analysis the *FanSmooth* (*r*=0.05) method. The value for spatial smoothness *r* has been determined after the empirical evaluation of obtained brain maps.

#### 3.2.1. Experiments Using Auditory EEG Data

In this section, we perform experiments using EEG data that corresponds to an auditory oddball paradigm and they can be downloaded from the homepage of the FieldTrip toolbox2. The raw EEG data consist in 600 trials. The duration of each trial was 2 secs, 1sec of EEG data preceding the acoustic stimulus, and 1sec of EEG data following the stimulus. The EEG activity was recorded using 128 channels at 1000 Hz. The EEG trials were band-pass filtered at 1–40 Hz and downsampled at 250 Hz. A realistic head model was used based on cortical surface approach. The number of dipoles was 5124; hence, the resulting lead field matrix is **H** ∈ *ℜ*^128×5124^. ERPs were formed by averaging over all trials. In this experiment, brain sources are detected by algorithms for the time point that corresponds to the peak of the electrical activity in the frontal-central scalp in the time range between 100 ms and 200 ms.

**Figure 3:**
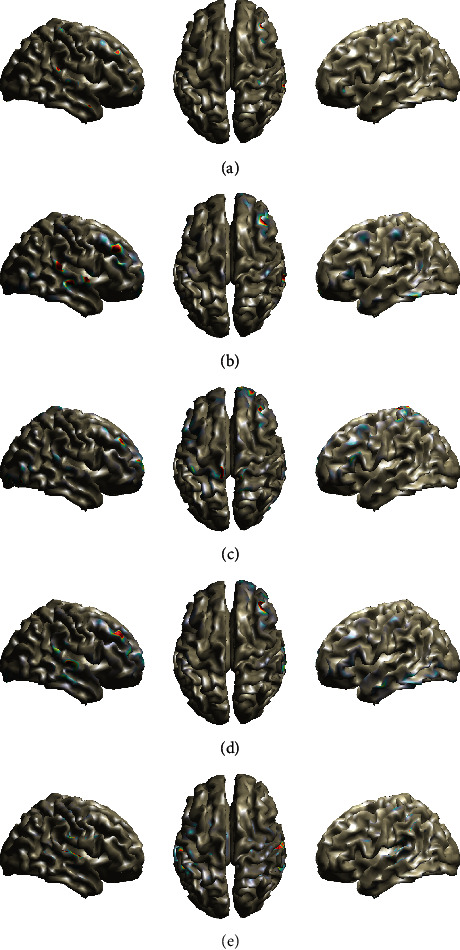
Brain maps showing EEG sources in the case of auditory EEG data. (a) Fan. (b) Fan smooth. (c) MNE. (d) RVM-VB. (e) Champ.

The estimated brain activity using the aforementioned methods is shown in Figure 3. The *Fan*, *FanSmooth*, *RVM-VB*, and *Champ* methods present activations in the temporal lobe, as expected in auditory experiments. However, the *Fan*, the *FanSmooth*, and the *Champ* methods provide activations on both hemispheres of the temporal lobe, while the *RVM-VB* method provides activations only to the right temporal lobe. The *MNE* method does not show activation in the temporal lobe. In addition to the above, we observe that all methods, besides *Champ*, present activations in the right frontal lobe. This type of activation is not unusual in auditory experiments, especially when deviant tones are involved [35, 36].

#### 3.2.2. Experiments Using Visual (Facial) Evoked Potentials EEG Data

The EEG data used in this section is part of the Multimodal Face Dataset available in the SPM software 3. This dataset was acquired from a face perception study in which the subject had to judge the symmetry of a mixed set of faces and scrambled faces. More details about the dataset can be found in [37]. The EEG acquisition system was a 128-channel ActiveTwo Biosemi system with a sampling frequency equal to 2048 Hz. The data were downsampled to 256 Hz, and after artifact rejection, the 309 epochs were averaged and low-pass filtered at 20 Hz. A realistic head model was used based on cortical surface approach. The number of dipoles was 5124; hence, the resulting lead field matrix is **H** ∈ *ℜ*^128×5124^.

**Figure 4:**
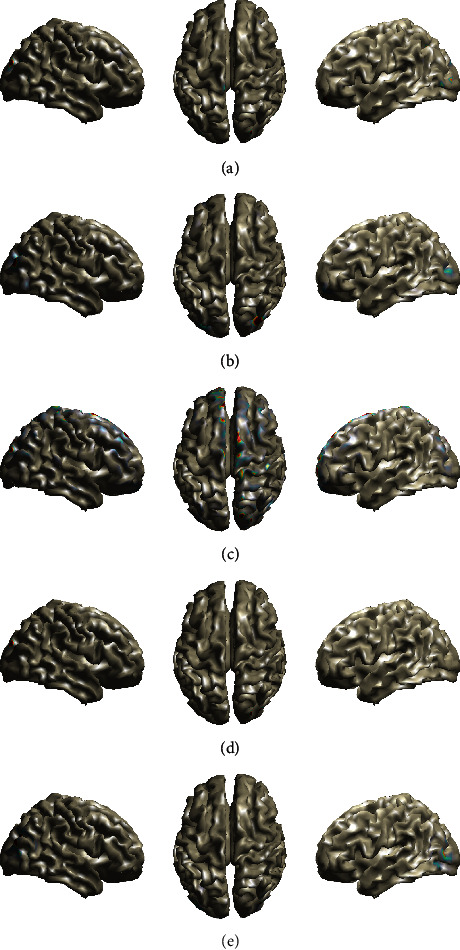
Brain maps showing EEG sources in the case of Visual (Faces) EEG data. (a) Fan. (b) Fan smooth. (c) MNE. (d) RVM-VB. (e) Champ.

The estimated activities from all methods are shown in Figure 4 (at 100 ms). Careful inspection of these images reveals that all methods present their primary activations on the occipital lobe as expected in this kind of experiment. However, we can also observe substantial differences with respect to the type of activation. More specifically, the *RVM-VB* and the *Fan* methods present the most compact activated area compared to other methods. Additionally, the *Fan*, the *FanSmooth*, and the *Champ* methods present bilateral activation on the occipital lobe, while the *RVM-VB* and the *MNE* methods present activation only to the right occipital lobe. Furthermore, the *Fan* and the *MNE* methods present secondary activations on the frontal lobe. In addition to that, the *MNE* method presents activations to the Supplementary Motor Area.

#### 3.2.3. Experiments Using Steady-State Visual Evoked Potentials EEG Data

In this subsection, the EEG data corresponds to a Steady-State Visual Evoked Potentials (SSVEP) Brain-Computer Interface (BCI) paradigm [38]. In this dataset, 40-target visual stimuli were presented on a 23.6 in LCD monitor. Thirty-five healthy subjects with normal or corrected-to-normal vision participated in this study. EEG data were recorded with 64 electrodes according to an extended 10–20 system in order to record whole-head EEG. Data epochs were extracted according to event triggers generated by the stimulus program. All data epochs were downsampled to 250 Hz. The EEG data have been band-pass (zero phases) filtered from 4 Hz to 90 Hz with an infinite impulse response (IIR) filter (by using the *filtfilt* function in MATLAB). From this dataset for our analysis, we have used the EEG trials from the first subject which are corresponding to the first target.

**Figure 5:**
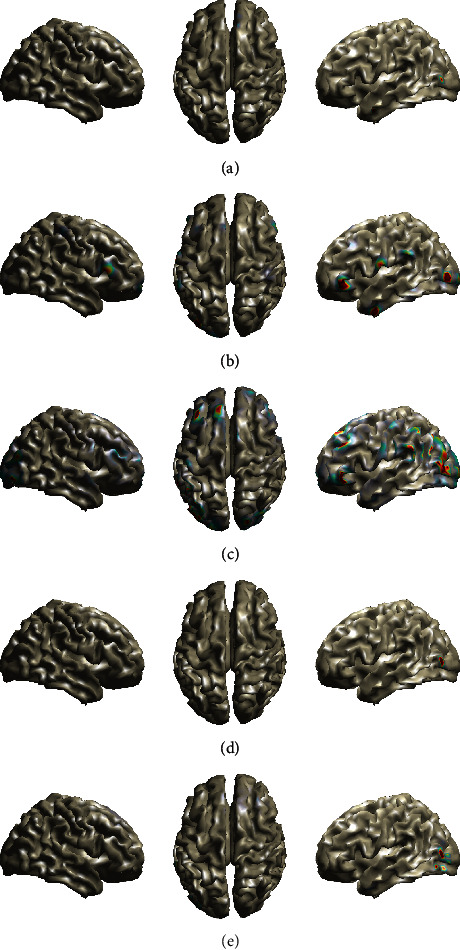
Brain maps showing EEG sources in the case of SSVEP EEG data. (a) Fan. (b) Fan smooth. (c) MNE. (d) RVM-VB. (e) Champ.

In this experiment, brain sources are detected by calculating the average scalp electrical activity between 1 sec and 4 sec. The estimated brain activity for all algorithms is shown in Figure 5. We can observe that all algorithms provide activated areas in the left part of the occipital lobe. In addition to that, the *MNE* methods provide also activations on the right part of the occipital lobe. Furthermore, we can observe activations on the frontal lobe from *FanSmooth* and *MNE* methods, while the *Fan* and *FanSmooth* methods provide an additional activation on the temporal lobe.

Concluding this section with real EEG data, it is worth providing a qualitative comparison between the methods and their properties. The *Fan* algorithm provides the most compact activated areas compared to other methods due to their inherent characteristic of sparseness. This observation is justified by observing the results when real EEG data are used as well as the “theoretical” implications of equation (3). On the other side, the *FanSmooth* algorithm provides a spatially extended activated area. Between these two extreme cases lie the *RVM-VB* algorithm and the *Champ* algorithm. However, this was expected due to the fact that (1) the *RVM-VB* algorithm and the *Champ* algorithm use a similar prior for EEG sources, which does not encourage sparsest solutions than our proposed prior, and (2) the basic version of them cannot handle spatially extended sources.

#### 3.2.4. Volumetric Lead Field Matrix

In this section, we provide experiments using the Faces EEG data. However, we have used a volumetric lead field matrix where the dipoles are placed on a spatial grid covering the entire brain. The grid resolution was set to 1 cm resulting in 2020 dipoles; hence, the resulting lead field matrix is **H** ∈ *ℜ*^128×(3 × 2020)^. Our goal in these experiments is to explore the behavior of our algorithms when groups of elementary dipoles are present. We perform a comparison between *FanGr*, *Fan*, and *Champ* algorithms. The *FanGr* algorithm is an extension of *Fan* algorithm when we want to utilize groups of dipoles, while we have used *Champ* algorithm as a baseline algorithm for comparative purposes.

**Figure 6:**
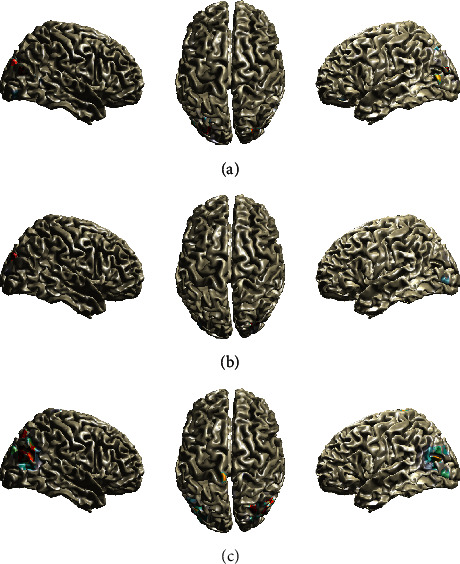
Brain maps (projected on the cortical surface) showing EEG sources in the case of Visual (Faces) EEG data when a volumetric-based lead field matrix is used. (a) Fan. (b) FanGr. (c) Champ.

In Figure 6, we provide the estimated activity of the aforementioned algorithms for the Faces EEG data. The preprocessing steps of EEG data are described in Section 3.2.2. We can observe that all algorithms provide activation in the occipital lobe as expected. However, we can observe differences in the pattern of activations. The activated area is larger in the *Champ* algorithm, followed by *Fan* algorithm, and, lastly, the *FanGr* algorithm provides the smallest activated area in the occipital lobe. We can, also, observe that the strength of activation is stronger in the left part of the occipital lobe for the *FanGr* and *Champ* algorithms, while the *Fan* algorithm presents strong activations on both parts of the occipital lobe. In addition to the above, we can observe that the *Champ* algorithm provides a secondary activation in the parietal lobe which cannot be justified by the type of experiments and the results that we obtained by all other algorithms and the two lead field matrices; hence, we assume that this activation is a spurious one. Concluding this section, we want to mention that both types of lead field matrices do not affect considerably the obtained results, irrelevant to the method that it was used to solve the inverse EEG problem. However, this observation is also affected by the type of EEG experiment.

## 4. Conclusions

In this work, we proposed a new algorithm (and its gradual extensions) to solve the EEG inverse problem. In this type of inverse problems, crucial part has the regularization term. In order to regularize the EEG inverse problem, we adopt the Bayesian approach; hence, regularizations are incorporated into the overall procedure in terms of prior distributions. Furthermore, we proposed new sparse priors for the modelling of EEG sources. The main contribution of these priors is that now we are able to examine the notion of sparseness in EEG source modelling, using structures of groups. Additionally, the basic idea of CS framework was used to provide us with modified lead field matrices specialized in modelling spatially extended EEG sources. Under the Bayesian formulation, the posterior distribution in our problem was intractable and to figure out this problem, we adopted the VB framework. The proposed Bayesian methods have been tested using head models with different geometries. The obtained results, using synthetic and real EEG data, show the merits of our methods in the estimation of EEG sources.

In the future, our research will be focused on accurate modelling of the head's properties and spatiotemporal extensions of our method with applications in the BCI domain [39–41]. More specifically, we intend to combine head models with different head geometries and tissue conductivities by adopting the multikernel learning methodology. The multikernel approach could lead us to the simultaneous estimation of the extended (or composite) lead field matrix and the EEG sources in an iterative fashion. Furthermore, spatiotemporal versions of our model based on the MMV model [1, 2, 19] could be devised in order to study EEG microstates [42] in BCI domain. In addition to the above, borrowing ideas from image superresolution [43], we could provide brain imaging techniques with increased spatial resolution. Finally, the EEG source localization has close connections with CS theory [30, 44]. However, typical approaches on the construction of lead field matrix do not produce a sensing matrix with the two basic properties of CS theory, the incoherence and the restricted isometry property. It is important to investigate procedures that could provide us with a lead field matrix that possesses these two properties.

## Data Availability

Auditory EEG data had been found at http://www.fieldtriptoolbox.org. Face Perception EEG data had been found at https://www.fil.ion.ucl.ac.uk/spm/data. SSVEP EEG data are freely available from http://bci.med.tsinghua.edu.cn/download.html.
